# Asymmetric dynamic interaction shifts synchronized frequency of coupled oscillators

**DOI:** 10.1038/s41598-020-58854-2

**Published:** 2020-02-13

**Authors:** Seong-Gyu Yang, Hyunsuk Hong, Beom Jun Kim

**Affiliations:** 1 Sungkyunkwan University, Department of Physics, Suwon, 16419 Republic of Korea; 2 Jeonbuk National University, Department of Physics and Research Institute of Physics and Chemistry, Jeonju, 54896 Republic of Korea

**Keywords:** Statistical physics, thermodynamics and nonlinear dynamics, Applied mathematics

## Abstract

Interacting dynamic agents can often exhibit synchronization. It has been reported that the rhythm all agents agree on in the synchronized state could be different from the average of intrinsic rhythms of individual agents. Hinted by such a real-world behavior of the interaction-driven change of the average phase velocity, we propose a modified version of the Kuramoto model, in which the *i*th oscillator of the phase *ϕ*_*i*_ interacts with other oscillator *j* only when the phase difference ${{\phi }}_{{j}}$ − ${{\phi }}_{i}$ is in a limited range [−*βπ*, *απ*]. From extensive numerical investigations, we conclude that the asymmetric dynamic interaction characterized by *β* ≠ *α* leads to the shift of the synchronized frequency with respect to the original distribution of the intrinsic frequency. We also perform and report our computer-based synchronization experiment, which exhibits the expected shift of the synchronized frequency of human participants. In analogy to interacting runners, our result roughly suggests that agents tend to run faster if they are more influenced by runners ahead than behind. We verify the observation by using a simple model of interacting runners.

## Introduction

Synchronization is a widespread phenomenon and has been observed in various physical and biological systems like lasers, sperms, fireflies, and so on^1–6^. Winfree proposed a model of coupled oscillator system^7,8^ and Kuramoto simplified the model later to make it analytically tractable^9,10^. Kuramoto model has also been studied in a variety of different interaction structures, including *d*-dimensional regular lattices and complex network structures^11–13^.

There have been various extensions since the original Kuramoto model has been proposed. For example, the effect of the time delay in interaction between oscillators has been studied^14,15^, and the correlation between the characteristic frequency and the coupling strength to describe the neural activity in brain has been investigated^16–18^. However, most existing studies have assumed that the interaction structure is static and thus does not change in time. Furthermore, extension of the Kuramoto model in such a way that the dynamic states of the oscillators are closely coupled with interaction structure has not been tried. In reality, it is often observed that the interaction structure can be dynamically coupled with the internal state of individual agent, and thus the oscillator system can hardly be an exception. In our approach in this paper, the asymmetric dynamic interaction due to the difference of phases of oscillators is proposed, which has not been considered in existing studies. We believe that our proposed model is also realistic and perform computer-based experiments of human participants.

The synchronized rhythm of oscillators can be faster or slower than average frequency of isolated individual oscillators. For example, music play of finger tapping has been reported to show a faster paired synchronized rhythm when there is no timing cue by the director^19^. The hand clapping of students has also been studied and faster synchronized rhythm like for the finger tapping^19^ has been reported^20^. For applause of audiences, however, slower synchronized rhythm has been observed^21^. Although a broad range of phenomena can be described through the use of the Kuramoto model, above mentioned behavior of the off-the-mean synchronized rhythm cannot be explained by the conventional model. One can easily understand this by a simple symmetry argument: The equation of motion for the conventional Kuramoto model is invariant under the phase reversal transformation ${\phi }_{i}\to -{\phi }_{i}$ for ∀*i*, and thus the shift of the average phase velocity $\langle {\dot{\phi }}_{i}\rangle$ contradicts the symmetry. From this reason, we suggest that we need to break the phase reversal symmetry in the conventional Kuramoto model in order to explain the observed shift of the synchronized rhythm. In our model, the phase reversal symmetry is broken not explicitly, but dynamically, as will be explained below. Furthermore, we perform smartphone and computer-based experiments with human participants to benchmark the model study and the results of the experiment supports our model which results in the off-the-mean frequency.

## Model

**Figure 1:**
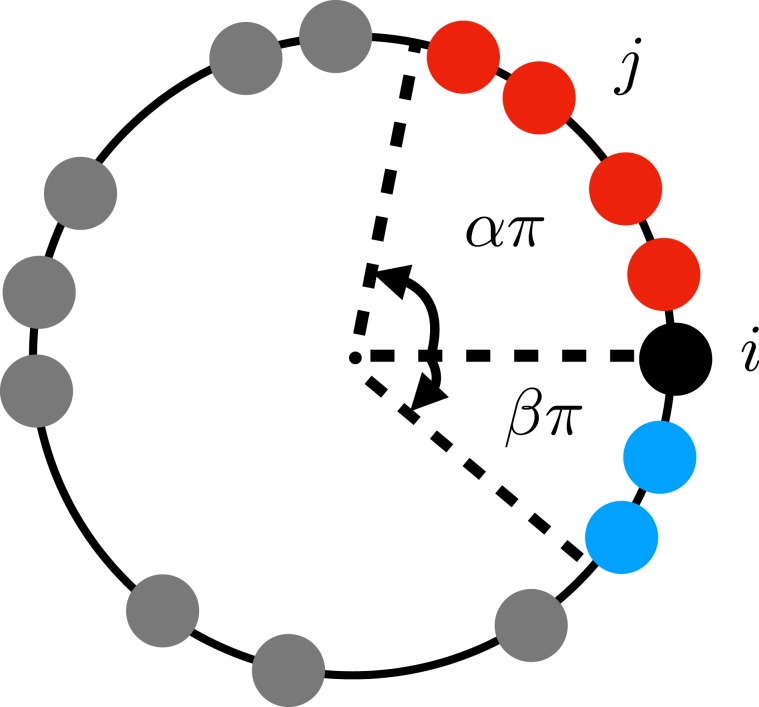
Schematic diagram for the interaction structure. The black filled circle denotes the *i*th oscillator with the phase ${\phi }_{i}(t)$, and the red (blue) ones the oscillators with phases ahead (behind) of ${\phi }_{i}(t)$. The interaction set ${\Lambda }_{i}(t)$ for the *i*th oscillator contains the oscillators denoted as red and blue filled circles with $|{\Lambda }_{i}(t\mathrm{)|=6}$ [see Eq. (2)]. All the other gray ones are the oscillators outside of the interaction range. We call the interaction symmetric when $\alpha =\beta$, and asymmetric when $\alpha \ne \beta$. The conventional Kuramoto model corresponds to the symmetric case with $\alpha =\beta =1$.

We suggest a simple modified Kuramoto model of *N* coupled oscillators with dynamic interaction to mimic the shift of the average frequency:

1 $${\dot{\phi }}_{i}={\omega }_{i}+\frac{K}{|{\Lambda }_{i}(t)|}\sum _{j\in {\Lambda }_{i}(t)}\sin ({\phi }_{j}-{\phi }_{i}),\,i=\mathrm{1,}\,\cdots ,N,$$

where $K\mathrm{( > 0)}$ is the coupling strength, and ${\phi }_{i}\in [-\,\pi ,\pi )$ and *ω*_*i*_ are the phase variable and the time-fixed (quenched) intrinsic frequency of the *i*th oscillator, respectively. The intrinsic frequency *ω*_*i*_ is chosen randomly from the normal distribution $g(\omega )=\mathrm{(1/}\sqrt{2\pi })\exp (\,-\,{\omega }^{2}\mathrm{/2)}$ with zero mean and unit variance. In detail, we first generate *N* random numbers ${\omega {\prime} }_{i}$ from the normal distribution and use the shifted frequency ${\omega }_{i}={\omega {\prime} }_{i}-\frac{1}{N}\sum {\omega {\prime} }_{i}$ as the intrinsic frequency in Eq. (1) to make it sure that the average intrinsic frequency $\frac{1}{N}\sum {\omega }_{i}$ is null. The null value of the mean frequency suggests that all phase variables and phase velocities are to be measured in the center of mass frame in which $\sum {\omega }_{i}=0$. Likewise, ${\dot{\phi }}_{i}=0$ does not mean that the oscillator is not oscillating but it only means that it is oscillating with the average intrinsic frequency. Our model differs from the conventional Kuramoto model in that the interaction structure dynamically depends on phase variables: ${\Lambda }_{i}(t)$ in Eq. (1) is the set of oscillators which the *i*th oscillator interact with and is written as

2 $${\Lambda }_{i}(t)=\{j|-\beta \pi \le {\phi }_{j}(t)-{\phi }_{i}(t)\le \alpha \pi \},$$

where ${\phi }_{j}(t)-{\phi }_{i}(t)$ is defined modulo 2*π*. Our key parameters are *α* and *β* in ${\Lambda }_{i}(t)$, which determine the condition for interaction. It is to be noted that if *α* = *β* = 1, all oscillators interact with all other oscillators and thus our model becomes identical to the globally-coupled Kuramoto model. If *α* = *β* < 1, the interaction becomes limited but is still symmetric. If *α* > *β* (*α* < *β*), on the other hand, the interaction becomes asymmetric toward oscillators with advanced (lagged) phases. The two asymmetric cases are equivalent to each other under the phase reversal transformation, ${\phi }_{i}\to -{\phi }_{i}$ for ∀*i* and $\alpha \leftrightarrow \beta$. Accordingly, we only focus on two cases: The symmetric interaction with *α* = *β* and forward-biased asymmetric interaction with *α* > *β*. In Fig. 1, we display a schematic diagram for the meaning of *α* and *β* in Eq. (2).

### Numerical results

We numerically integrate Eq. (1) using the Heun’s method, which is the second-order algorithm, with the discrete time step $\Delta t=0.01$ for $2\times {10}^{5}$ time steps, corresponding to $t\in \mathrm{[0,}\,\mathrm{2000]}$. After a sufficiently long time, the system arrives at the steady state, and the first half ${10}^{5}$ time steps are discarded before we start to measure observables for the later half time steps. All the results in the present paper are obtained from the averages over 100 (200 for $N=25$) independent and different random initial phase variables and intrinsic frequency realizations.

We measure the standard order parameter *R* for the synchronization transition defined as

3 $$R\equiv \langle \bar{|\frac{1}{N}\mathop{\sum }\limits_{j}^{N}{e}^{i{\phi }_{j}(t)}|}\rangle ,$$

where $\langle \cdots \rangle$ and $\overline{\cdots }$ denote the ensemble and the temporal averages, respectively. We also measure the average phase velocity Ω in the steady state defined as

4 $$\Omega \equiv \langle \overline{\frac{1}{N}\mathop{\sum }\limits_{i}^{N}{\dot{\phi }}_{i}(t)}\rangle .$$

**Figure 2:**
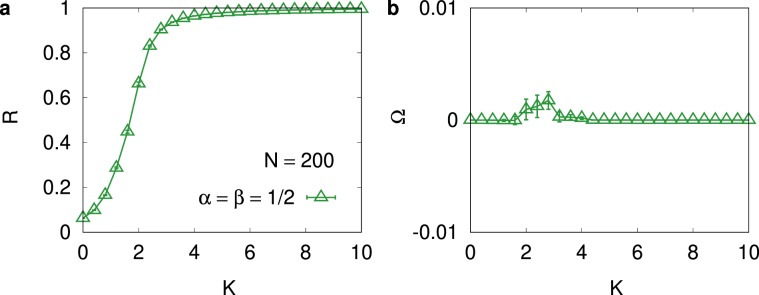
Numerical results for symmetric case $\alpha =\beta =\mathrm{1/2}$. (**a**) The order parameter *R* and (**b**) the average phase velocity Ω versus the coupling strength *K* for the system size $N=200$ when the interaction is symmetric $(\alpha =\beta =\mathrm{1/2})$. *R* in (**a**) clearly indicates the existence of the synchronization transition around ${K}_{c}\approx 2$ and Ω in (**b**) remains close to zero irrespective of *K*.

We first present our result for the symmetric case that $\alpha =\beta =\mathrm{1/2}$ in Fig. 2 for $N=200$. Results for other symmetric cases $\alpha =\beta =1/4,3/4$, and 1 exhibit qualitatively the same behavior and thus are not presented here. From Fig. 2a, we notice that the synchronization order parameter *R* in Eq. (3) clearly exhibits the synchronization transition around the transition point ${K}_{c}\approx 2$, which separates the asynchronous $(R\approx 0)$ phase for $K < {K}_{c}$ and the synchronous $(R={\mathscr{O}}\mathrm{(1)})$ phase for $K > {K}_{c}$. In contrast, at any value of *K*, the average phase velocity Ω in Eq. (4) remains close to 0. A small bump in Fig. 2b around $K\approx {K}_{c}$ does not depend much on the system size, but it becomes shallower as the number of samples used in the ensemble average is increased. We believe that the bump structure is a reminiscent of the large fluctuation of the phase velocity around the critically. We thus conclude that the symmetric interaction characterized by $\alpha =\beta \, > \,0$ does not change the average phase velocity from the average intrinsic frequency, which is set to null, irrespective of whether the system is in the asynchronous or in the synchronous phase.

Our observation of null average phase velocity for $\alpha =\beta$ can be understood through a simple symmetry argument: Due to the phase reversal symmetry under the transformation ${\phi }_{i}\to -{\phi }_{i}$ for ∀_*i*_ for the symmetric case of $\alpha =\beta$, there is no reason that the phase velocity is biased toward a positive or a negative value. Consequently, the average phase velocity Ω must be identical to the average intrinsic frequency $\langle \frac{1}{N}{\sum }_{i}{\omega }_{i}\rangle$, which is set to null as described above. In contrast, the off-the-mean synchronized rhythm has been often reported in music play and hand clapping^19–21^. Accordingly, we suggest that such observation of the shift can be explained by the biased (or asymmetric) interaction.

**Figure 3:**
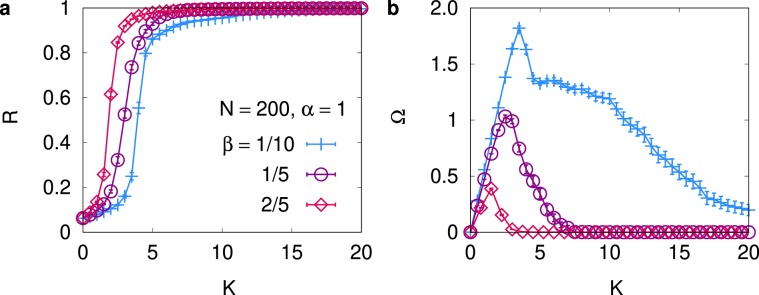
Numerical results for several asymmetric cases. (**a**) *R* and (**b**) Ω versus *K* for $N=200$ when the interaction is asymmetric ($\alpha =1$ and $\beta =1/10,1/5,2/5$). The average phase velocity Ω in (**b**) clearly displays the shift from the average intrinsic frequency $\langle \omega \rangle =0$, but the shift becomes negligible in the limit of the strong coupling.

For the asymmetric cases $(\alpha  > \beta )$, we first display our results for $\alpha =1$ and $0\, < \,\beta \, < \,1$ in Fig. 3 for $N=200$. As shown in Fig. 3(a), the system again exhibits a well-defined synchronization transition and the order parameter *R* changes from $R\approx 0$ to $(R={\mathscr{O}}\mathrm{(1)})$ as the coupling strength *K* is increased. The average phase velocity Ω in Fig. 3b, in contrast, shows a very interesting behavior, which sharply differs from what has been observed for the symmetric interaction in Fig. 2. As *K* is increased from null value, Ω first increases with *K*, and displays a peak, beyond which Ω decreases with *K*, as shown in Fig. 3b. The observed nonmonotonous behavior of Ω in Fig. 3b can be understood as follows: In the limit of the vanishing coupling strength $K\to 0$, all oscillators are completely decoupled and the phase reversal symmetry is preserved to yield Ω = 0. As *K* is increased, the asymmetric interaction breaks the symmetry and Ω increases. In the opposite limit of the strong coupling $(K\to \infty )$, all oscillators are fully synchronized and interact with all other oscillators as in the globally-coupled Kuramoto model. Consequently, we expect $\Omega \to 0$ in both limits of $K\to 0$ and $K\to \infty$, which suggests that the existence of a peak of Ω in the middle is inevitable. It appears that the peak position of Ω is close to the synchronization transition point in Fig. 3a. Although not shown here, we also check the finite-size effect on the shift of Ω, only to find that the peak structure becomes more enhanced in a larger system. We thus conclude that our modified Kuramoto model with asymmetric interaction reveals the observed off-the-mean synchronized rhythm in previous studies^19,20^.

**Figure 4:**
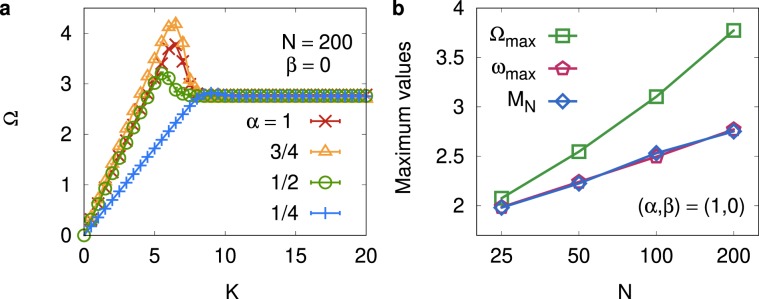
Numerical results for limiting case of asymmetric interaction. (**a**) The average phase velocity Ω versus *K* is shown for $\beta =0$ and $\alpha =1,3/4,1/2,1/4$ for $N=200$. Ω in (**a**) exhibits a peak around the synchronization transition and the plateau in the strong-coupling regime. (**b**) Peak height Ω_max_ in (**a**), the maximum intrinsic frequency ${\omega }_{{\rm{\max }}}={{\rm{\max }}}_{i}{\omega }_{i}$, and its analytic value ${M}_{N}$ (see text) for $(\alpha ,\beta )=\mathrm{(1,0)}$ versus the system size *N*. ${\Omega }_{{\rm{\max }}}$ is larger than both ${M}_{N}$ and ${\omega }_{{\rm{\max }}}$, but follows the similar form of the logarithmic increase.

We next investigate the limiting case of the asymmetric interaction characterized by *α* > 0 and $\beta =0$. In words, this type of interaction means that each oscillator interacts with other oscillators only when their phases are ahead. Figure 4a displays the average phase velocity Ω for $\beta =0$ and $\alpha =1,3/4,1/2$, and $\mathrm{1/4}$ for $N=200$. We find that the decrease of Ω in the strong-coupling limit observed in Fig. 3b disappears for $\beta =0$, and Ω stays at a positive value even when $K$ is increased to a very large value. We emphasize that the strong-coupling limit for $\beta =0$ is different from the same limit for $\beta \ne 0$: The interaction cannot be of all-to-all type for the former case due to the strict bound $\beta =0$. We thus expect that the finite positive value of Ω should persist even when the coupling strength $K\to \infty$.

Similarly to the peak structure in Fig. 3b for $\beta  > 0$, the peak of Ω is clearly seen in Fig. 4a for $\beta =0$, near the synchronization transition point. We believe that the height of the peak of Ω can be related with maximum intrinsic frequency. The maximum value ${M}_{N}$ of *N* random numbers generated from the normal distribution is given by ${M}_{N}={m}_{N}+\gamma {m}_{N}\mathrm{/(1}+{m}_{N}^{2})$ with ${m}_{N}\equiv {[\mathrm{ln}({N}^{2}\mathrm{/2}\pi )-\mathrm{ln}\mathrm{ln}({N}^{2}\mathrm{/2}\pi )]}^{\mathrm{1/2}}$ and the Euler’s constant $\gamma$^22,23^. In Fig. 4b, we display the peak height ${\Omega }_{{\rm{\max }}}$ [see Fig. 4a], the measured value of ${\omega }_{{\rm{\max }}}={{\rm{\max }}}_{i}\,{\omega }_{i}$, and ${M}_{N}$, versus $N$ for $(\alpha ,\beta )=\mathrm{(1,}\,\mathrm{0)}$. The latter two values, ${\omega }_{max}$ and ${M}_{N}$, almost coincide as expected, and ${\Omega }_{{\rm{\max }}}$ follows the similar form of the logarithmic increase. However, the peak height ${\Omega }_{{\rm{m}}{\rm{a}}{\rm{x}}}$ is significantly larger than ${M}_{N}$ (e.g, ${\Omega }_{max}/{M}_{N}\approx 1.36$ for $N=200$), which means that the average phase velocity of oscillators is larger than the maximum intrinsic frequency. We believe that this result is particularly interesting, since the forward-only asymmetric interaction drives all oscillators to have angular velocity beyond the maximum intrinsic frequency. In contrast, the peak height in Fig. 3b for $\beta =\mathrm{1/10}$ is found to be less than the maximum intrinsic frequency (we find ${\Omega }_{max}/{M}_{N}\approx 0.68)$.

**Figure 5:**
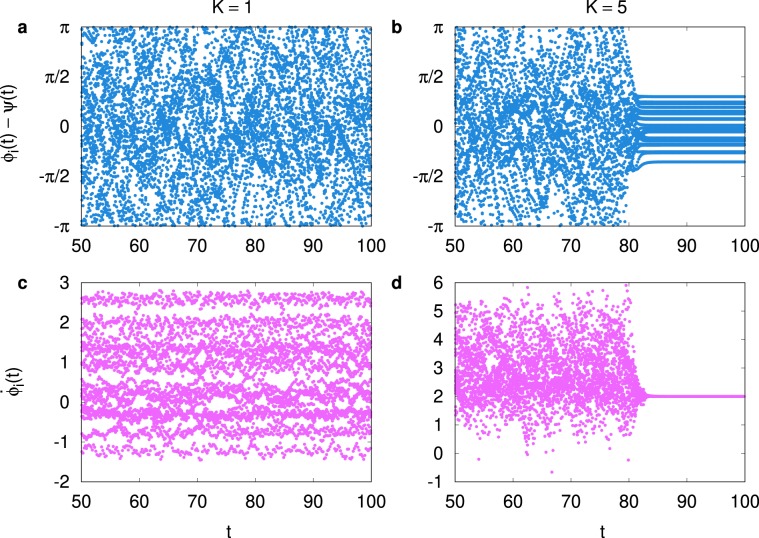
The phase trajectories [(**a**,**b**)] and the phase velocities [(**c**,**d**)] of $N\mathrm{=25}$ individual oscillators versus time *t* for $(\alpha ,\beta )=\mathrm{(1,0)}$ and for $K=1$ [(**a**,**c**)] and for $K=5$ [(**b**,**d**)]. We plot the trajectories [(**a**,**b**)] in the form of ${{\phi }}_{{i}}(t)-\psi (t)$ with *ψ*(*t*) being the phase of the synchronization order parameter (see the text). When the synchronization occurs in (**d**) for $K=5$ beyond *t* ≳ 80 after an initial transient, all the oscillators agree on the phase velocity close to the maximum intrinsic frequency ${\omega }_{max}\approx 2$.

In order to examine details of the dynamics of the system, we also integrate Eq. (1) starting from a single random initial condition and a single configuration of $\{{\omega }_{i}\}$ for $(\alpha ,\beta )=\mathrm{(1,0)}$ at coupling strengths $K=1$ and 5. In Fig. 5a,b we display individual phase trajectories for $N=25$ oscillators in the moving reference frame of the phase $\psi (t)$ of the order parameter defined by $R(t){e}^{i\psi (t)}\equiv \frac{1}{N}{\sum }_{i}{e}^{i{\phi }_{i}(t)}$. When the system is in the asynchronous phase at $K=1$, the phase trajectories ${\phi }_{i}(t)-\psi (t)$ do not exhibit any correlated behavior as shown in Fig. 5a. On the other hand, at $K=5$ near the synchronization transition point, trajectories become flat after some transient behavior as shown in Fig. 5b. We also display the individual phase velocity ${\dot{\phi }}_{i}(t)$ in Fig. 5c,d for $K=1$ and 5, respectively. The behavior reported in Fig. 5d for $K=5$ is particularly interesting since it shows how oscillators become synchronized in time. Before the system approaches the synchronized state, phase velocities are very different and scattered. It needs to be noticed that in this early transient stage, some oscillators have very large phase velocities $(\,\approx \,6)$ before synchronization. In the later synchronized state $(t\gtrsim 80)$, all oscillators agree on the frequency ${\dot{\phi }}_{i}\approx 2$, which is close to the maximum intrinsic frequency ${\omega }_{{\rm{\max }}}\approx 2$. In words, the final synchronized state is approached through a transient state in which some oscillators can have very large phase velocity.

### Experiments

We next perform smartphone and computer-based experiments with 31 individual human participants. The server computer plays the periodic sound of a metronome for 15 seconds and each participant is asked to press the touchpad(display) button of her/his notebook computer(android phone) in accord with the metronome sound. After 15 seconds, the metronome sound of the server is stopped, but each participant is asked to keep pressing the button for about 1 minute. We use two different programs in the client side to examine the effect of interaction among participants: (i) Without Interaction: The beeping sound of the client device is turned off. Accordingly, each player tries only to continue the rhythm of metronome (s)he listened for the first 15 seconds, without any interaction or interference with other participants. In this case, the interaction between players is unlikely since there is no sound cue in the later stage after 15 seconds and we ask players only to look at their computer or smartphone screens. (ii) With Interaction: The beeping sound is turned on, and all participants are asked to synchronize the beeping sound (s)he generates to the sound generated by other players. The advantage of using our platform is that the time instants of button pressing are automatically recorded in the server, which makes further analysis straightforward.

**Figure 6:**
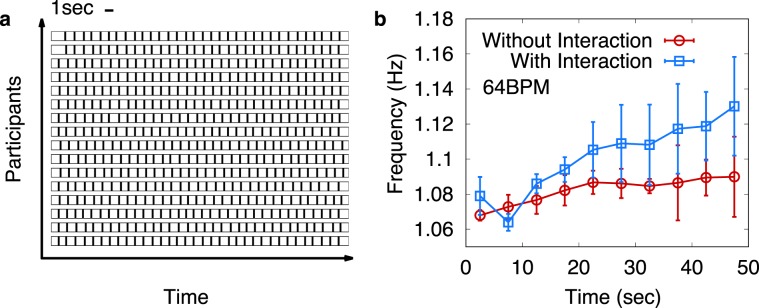
Experimental results for 31 participants. (**a**) Time instants of button pressings of players are displayed in the form of short vertical bars for 64 BPM metronome experiment with interaction. (**b**) The frequencies for individual players are measured with the time window of the size 5 seconds, and then are averaged over all players. We repeat the experiment four times for the 64 BPM metronome for (i) Without and (ii) With Interaction cases (see text). When the players are interacting with each other, the synchronized frequency in later time is found to be significantly larger than the metronome frequency 64 BPM(=1.07 Hz).

We use 64 and 120 BPM (beats per minute) for the metronome rhythm, which correspond to the metronome frequency 1.07 and 2.0 Hz, respectively. In Fig. 6a, we display partial results of our experiment for the 64 BPM metronome for the case of (ii) With Interaction, as an example. The time instants of button pressing are displayed in the form of the short vertical bars, as often used for the visualization of the neuron firing pattern in neuroscience^24^. In Fig. 6b, we show how the average frequency changes in time for the case of (i) Without and (ii) With Interaction. We compute the frequency of each individual by using the time window of 5 seconds, and results from four independent experiments are averaged. Note that the two curves almost coincide to the metronome frequency 1.07 Hz at around 10 second, before the metronome sound is stopped. As time goes on, it should be recognized that the average frequency for (ii) With Interaction case increases. Differently from previous study^19^, we have not observed downward shift of the synchronized frequency for (i) Without Interaction case. We believe that our experimental result in Fig. 6b supports our previous finding in Figs. 3 and 4, since the average frequency for (ii) With Interaction lies significantly higher than for (i) Without Interaction. However, for 120 BPM the difference between (i) Without and (ii) With Interaction case is found insignificant. Probably, 120 BPM could be too fast for some participants to comfortably follow.

### Interacting runners

We suggest that our main finding of the shift of the average phase velocity can also be tested by other more realistic phenomenon, like interacting runners. In this regard, it is interesting to note that researchers recorded two international marathon races and found the grouping behavior of the runners^25^. Furthermore, it has been found that the denser the group is, the faster the runners in the group are^25^, which indicates that the speed of a runner is not only an intrinsic property of the runner, but also can be an outcome from interaction with other runners.

Hinted by both the previous observation^25^ and our results for the shift of the average phase velocity of oscillators, we propose a simple model to mimic interacting runners in a one-dimensional linear track and write the equation of motion for the position ${x}_{i}$ of the *i*th runner as

5 $${\dot{x}}_{i}(t)={v}_{i}+\,min(C\,[\mathop{max}\limits_{j\in {\Lambda }_{i}(t)}{x}_{j}(t)-{x}_{i}(t)],{\sigma }_{v}),$$

where the characteristic speed ${v}_{i}$ is a Gaussian quenched random variable with the mean $\langle v\rangle =5\,{\rm{m}}/{\rm{s}}$ and the standard deviation ${\sigma }_{v}\mathrm{=1}\,{\rm{m}}/{\rm{s}}$, $C\mathrm{(=0.1/}{\rm{s}})$ is the competitive coupling strength, and ${\Lambda }_{i}(t)$ is the set of runners within the *i*th runner’s sight limit $\ell (\,=20\,{\rm{m}})$:

6 $${\Lambda }_{i}(t)=\{j\mathrm{|0}\le {x}_{j}(t)-{x}_{i}(t)\le \ell \mathrm{\}}.$$

**Figure 7:**
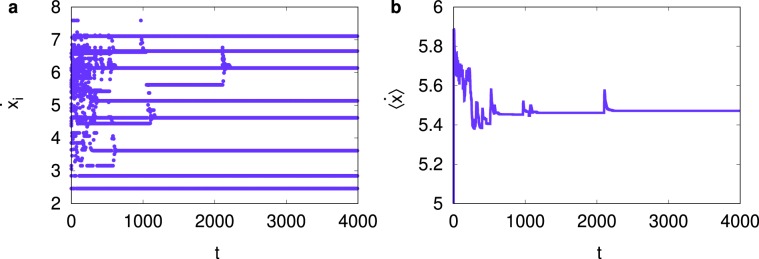
Numerical results for interacting runner model with competitive coupling strength $C\mathrm{=0.1}$ for system size $N\mathrm{=50}$. (**a**) Speeds $\{{\dot{x}}_{i}\}$ of individual runners versus time *t*. Runners start running at their own characteristic speed {*v*_*i*_} but as time goes on they form about 10 groups, in accord with the previous finding^25^. The size of each group is $\mathrm{4,8,14,8,9,5,1}$ and 1 in the order of group speed for *t* ≳ 2000. Runners within each group run at the same speed. (**b**) The average speed of all runners at later times is 5.5 m/s, which is larger than the average characteristic speed 5 m/s.

In Eq. (5), we try to mimic the situation that if one sees other runners in front in close distance, the runner tries to speed up within his limitation. However, if the distance to the front runner is too big, the runner may not try hard to catch up. Only for simplicity, we assume the runner’s speed is limited by ${v}_{i}+{\sigma }_{v}$ from that every human individual runner must have some physical and biological limitation. Note that if there is no front runner in the distance $\ell$, the runner runs at his intrinsic speed of ${v}_{i}$. Figure 7 shows our result for the forward-interacting runners for $N\mathrm{=50}$. We first observe that runners form groups of different speeds, and the runner’s speed within each group is identical. It is to be noted that although our model of runners with forward-biased interaction is very simple, it produces the grouping behavior reported in previous study^25^. The average speed of all runners is measured 5.5 m/s, which is larger than the average characteristic speed $\langle {v}_{i}\rangle \mathrm{=5}\,{\rm{m}}/{\rm{s}}$. The qualitative feature of the grouping behavior is observed robust if the value of *C* is not too small, nor too large. Although our interacting runner model does not include physiological factors which affect the performance of runners^26,27^, the shift of the average speed is observed as in the marathon race^28^. We emphasize that this finding of the shift of the average speed is in parallel to our observation for oscillators with asymmetric interaction in Figs. 3 and 4 as well as our experimental observation in Fig. 6.

## Discussion

In this paper, we have suggested models of interacting oscillators and runners, in which the key ingredient is the forward-biased interaction. Through numerical investigations, we have observed that such asymmetric interaction shifts the average velocity in the two model systems, in accord with the findings in previous studies^19,20^. Our simple model of forward-interacting runners has also been shown to produce the grouping behavior reported in previous study^25^. We have also performed a computer-based synchronization experiment of interacting human individuals and observed again the shift of the average frequency. We believe that when we synchronize sound rhythm with others we tend to catch up the sound of others slightly ahead in time. Accordingly, we suggest that our modified Kuramoto model can have some relevance in explaining our experimental result of sound synchronization of human individuals. The asymmetric dynamic interaction has not been studied before in the research field of synchronization and our new computer-based experiments also show that our model study is realistic.

## Methods

### Experiment participants

31 undergraduate students of Sungkyunkwan University participated in the experiment described in this manuscript voluntarily. The experiments approved by the institutional review board of Sungkyunkwan University and conducted in accordance with the Declaration of Helsinki. All participants provided informed consent.
